# Assessment of Variability in End-of-Life Care Delivery in Intensive Care Units in the United States

**DOI:** 10.1001/jamanetworkopen.2019.17344

**Published:** 2019-12-11

**Authors:** Jacqueline M. Kruser, David A. Aaby, David G. Stevenson, Brenda T. Pun, Michele C. Balas, Mary Ann Barnes-Daly, Lori Harmon, E. Wesley Ely

**Affiliations:** 1 Division of Pulmonary and Critical Care, Department of Medicine, Northwestern University, Chicago, Illinois; 2 Department of Medical Social Sciences, Northwestern University, Chicago, Illinois; 3 Division of Biostatistics, Department of Preventive Medicine, Northwestern University, Chicago, Illinois; 4 Department of Health Policy, Vanderbilt University, Nashville, Tennessee; 5 Geriatric Research, Education and Clinical Center Service, Department of Veterans Affairs Medical Center, Tennessee Valley Healthcare System, Nashville, Tennessee; 6 Critical Illness, Brain Dysfunction, and Survivorship Center, Vanderbilt University Medical Center, Nashville, Tennessee; 7 College of Nursing, The Ohio State University, Columbus; 8 Sutter Health, Sacramento, California; 9 Society of Critical Care Medicine, Mount Prospect, Illinois; 10 Division of Pulmonary and Critical Care, Department of Medicine, Vanderbilt University, Nashville, Tennessee

## Abstract

**Importance:**

Overall, 1 of 5 decedents in the United States is admitted to an intensive care unit (ICU) before death.

**Objective:**

To describe structures, processes, and variability of end-of-life care delivered in ICUs in the United States.

**Design, Setting, and Participants:**

This nationwide cohort study used data on 16 945 adults who were cared for in ICUs that participated in the 68-unit ICU Liberation Collaborative quality improvement project from January 2015 through April 2017. Data were analyzed between August 2018 and June 2019.

**Main Outcomes and Measures:**

Published quality measures and end-of-life events, organized by key domains of end-of-life care in the ICU.

**Results:**

Of 16 945 eligible patients in the collaborative, 1536 (9.1%) died during their initial ICU stay. Of decedents, 654 (42.6%) were women, 1037 (67.5%) were 60 years or older, and 1088 (70.8%) were identified as white individuals. Wide unit-level variation in end-of-life care delivery was found. For example, the median unit-stratified rate of cardiopulmonary resuscitation avoidance in the last hour of life was 89.5% (interquartile range, 83.3%-96.1%; range, 50.0%-100%). Median rates of patients who were pain free and delirium free in last 24 hours of life were 75.1% (interquartile range, 66.0%-85.7%; range, 0-100%) and 60.0% (interquartile range, 43.7%-85.2%; range, 9.1%-100%), respectively. Ascertainment of an advance directive was associated with lower odds of cardiopulmonary resuscitation in the last hour of life (odds ratio, 0.70; 95% CI, 0.49-0.99; *P* = .04), and a documented offer or delivery of spiritual support was associated with higher odds of family presence at the time of death (odds ratio, 1.95; 95% CI, 1.37-2.77; *P* < .001). Death in a unit with an open visitation policy was associated with higher odds of pain in the last 24 hours of life (odds ratio, 2.21; 95% CI, 1.15-4.27; *P* = .02). Unsupervised cluster analysis revealed 3 mutually exclusive unit-level patterns of end-of-life care delivery among 63 ICUs with complete data. Cluster 1 units (14 units [22.2%]) had the lowest rate of cardiopulmonary resuscitation avoidance but achieved the highest pain-free rate. Cluster 2 (25 units [39.7%]) had the lowest delirium-free rate but achieved high rates of all other end-of-life events. Cluster 3 (24 units [38.1%]) achieved high rates across all favorable end-of-life events.

**Conclusions and Relevance:**

In this study, end-of-life care delivery varied substantially among ICUs in the United States, and the patterns of care observed suggest that units can be characterized as higher and lower performing. To achieve optimal care for patients who die in an ICU, future research should target unit-level variation and disseminate the successes of higher-performing units.

## Key Points

**Question:**

Do intensive care units in the United States provide high-quality end-of-life care?

**Findings:**

In this cohort study of 1536 decedents within a national quality improvement collaborative, end-of-life care delivery varied widely between intensive care units. There were 3 mutually exclusive unit-level patterns of end-of-life care delivery observed, which suggest meaningful differences in the experience of dying for patients cared for in higher-performing and lower-performing units.

**Meaning:**

To improve care for all patients who die in an intensive care unit, future research should target unit-level variation and investigate the latent characteristics of high-performing units that promote high-quality end-of-life care.

## Teaser

This cohort study describes the structures, processes, and variability of end-of-life care delivered in intensive care units in the United States.

## Introduction

Approximately 20% of people who die in the United States are admitted to an intensive care unit (ICU) at or near the time of death.^1^ The provision of high-quality end-of-life (EOL) care is an integral professional responsibility of ICU clinicians.^2,3,4,5,6,7^ Regional and single-center studies have uncovered gaps in the quality of EOL ICU care in domains such as symptom control, patient-centered decision-making, and the provision of adequate spiritual support.^8,9,10,11,12,13,14,15,16,17^ However, it is unknown whether these findings represent the current delivery of EOL ICU care across the United States.

Prior work^18,19,20,21^ demonstrated variation among units in the delivery of life-sustaining treatments and cardiopulmonary resuscitation (CPR) at the EOL. Observed unit-level variation in EOL care is not fully explained by differences in patient preferences or characteristics,^18,22^ suggesting unit-level characteristics and practice patterns may have an important association with EOL care delivery.^23^ Beyond the delivery of CPR and life-sustaining treatments, families of decedents and clinicians in the ICU recognize additional, meaningful events that take place during death and dying in an ICU, such as the presence of family at the bedside and the avoidance of burdensome symptoms near death.^10,11,24,25^ Whether these fundamental features of EOL care also vary among units within the United States is unknown.

To achieve the best possible EOL ICU care, we need a better understanding of the existing structures, processes, and patterns of care that underlie EOL ICU care delivery and that influence patients’ and families’ experiences with death and dying. Therefore, the primary objective of this nationwide multicenter cohort study was to identify unit-level opportunities to improve EOL care delivery in ICUs across the United States.

## Methods

### Setting

The ICU Liberation Collaborative (ILC) was a quality improvement learning collaborative of 68 ICUs within 67 hospitals (with 1 hospital with 2 participating units) across the United States and Puerto Rico. The collaborative took place from January 2015 to April 2017; it was coordinated and sponsored by the Society of Critical Care Medicine and the Gordon and Betty Moore Foundation. The primary objective of the ILC was to disseminate an interprofessional approach to ICU symptom management known as the ABCDEF bundle. All participating units received the same concurrent exposure to in-person meetings, monthly video conferences, an online interactive forum, and coaching from expert faculty. A complete description of the ILC has been previously published.^26,27^

### Participants and Data Collection

Patient-level data were collected from the first 15 consecutive patients admitted per month in each unit during the collaborative. Patient-level data were also provided for 30 patients admitted to the unit in the 6 months before the collaborative. Collaborative data collection procedures excluded patients who died, were discharged, underwent active withdrawal of life-sustaining treatment, or were designated as comfort-care only within the first 24 hours of ICU admission. Local team members were responsible for collecting and entering deidentified patient-level data into the ILC database. The Research Electronic Data Capture (REDCap) platform was used for local data entry, transmission to the coordinating site, and data storage. Local team members were trained to collect data during a 1-hour video conference and followed a standard operating procedure and data definitions manual. Unit-level data were collected at the beginning of the collaborative from each unit’s clinical manager or medical director through a standardized instrument within REDCap. All adults who died during their initial ICU stay in the periods before and during the collaborative were included in this analysis. Patients with missing discharge status (eg, alive, deceased) were excluded. The Vanderbilt University Medical Center institutional review board served as the coordinating center institutional review board and granted the ILC expedited quality improvement project approval. All participating units acquired site-specific institutional review board evaluation and approval, when required. The data and variables collected for this study were included in the original ILC data collection procedures and elements presented to the institutional review boards, so no additional approval or consent was sought for this analysis. This report adheres to the Strengthening the Reporting of Observational Studies in Epidemiology (STROBE) reporting guideline for cohort studies.

### Structures, Processes, and Outcomes

Multiple consensus statements have established ICU-specific EOL quality measures based on bioethical principles, expert opinion, and limited observational data.^2,7,9,28^ Study measures were derived from these published quality measures and were organized according to the Donabedian structure, process, outcome model and the 6 key domains of EOL ICU care (Table 1).^2,8,29,30,31,32,33^

**Table 1.** Measures for Structures, Processes, and Outcomes of EOL Care in the ICU

Domains of EOL Care	Structure	Process	Outcome
Symptom management	EOL-specific protocol for symptom management or withdrawal of mechanical ventilation	Assessment of pain or delirium in the last 24 hours of life	Patient pain free or delirium free in last 24 hours of life^a^^,^^b^
Emotional and practical support of patients and families	Policy for open visitation	…	Family or significant person(s) present at time of death
Patient-centered and family-centered decision-making	…	Ascertainment of advance directive during hospitalization	Absence of CPR in last hour of life; extubation before death^c^
Spiritual support	…	Offer or delivery of spiritual support during ICU stay	…
Continuity of care	Policy for continuity of nursing services	…	…
Emotional and organization support for clinicians	Policy for structured clinician reflection opportunities	…	…

Abbreviations: CPR, cardiopulmonary resuscitation; ellipses, not applicable; EOL, end of life; ICU, intensive care unit.

^a^ Pain free was defined as no significant pain episodes in last 24 hours of life among patients with at least 1 pain assessment. Significant pain was indicated by a numerical rating score greater than 3, a Critical Care Pain Observation Tool score greater than 2, and/or a Behavioral Pain Score greater than 3.

^b^ Delirium free was defined as no episodes of delirium in the last 24 hours of life among patients with at least 1 delirium assessment. Delirium was indicated by a positive score on the Confusion Assessment Method for the ICU score or an Intensive Care Delirium Screening Checklist score greater than 4.

^c^ Absence of CPR and extubation do not necessarily represent patient-centered and family-centered decision-making, given that individual treatment preferences will vary. This patient-level measure is derived from population-level findings that absence of CPR and extubation are both associated with higher rating of the quality of death and dying by family members.^11,24^

The 5 structural measures in this study evaluated the presence of the following unit-level policies or protocols: (1) EOL-specific protocols for general symptom management; (2) EOL-specific protocols for symptom management during withdrawal of mechanical ventilation; (3) an open visitation policy, defined as a policy that permits family members and friends to spend time in the patient’s room regardless of the time of day; (4) a policy for the continuity of nursing services for patients with multiple-day stays in the ICU; and (5) a policy that supports a regular, structured opportunity for clinicians to reflect about the experience of caring for dying patients.^2,7,8,11,32,33^ The 4 patient-level process measures were as follows: (1) at least 1 pain assessment documented in the electronic health record (EHR) in last 24 hours of life; (2) at least 1 delirium assessment documented in the EHR in the last 24 hours of life; (3) EHR documentation of the presence or absence of an advance directive (AD), health care power of attorney document, or living will at any time during the terminal hospitalization; and (4) EHR documentation that spiritual support was offered or delivered at any time during the ICU stay.^2,7,8,11,32,33^

We measured the outcomes of EOL ICU care through specific, meaningful EOL events that are associated with higher quality of death and dying in the ICU, as determined by decedents’ families and ICU clinicians.^10,11,24,25^ Patient-level EOL events were as follows: (1) extubation or discontinuation of invasive mechanical ventilation before time of death among patients receiving mechanical ventilation; (2) absence of CPR in the last hour of life; (3) being delirium free in the last 24 hours of life; (4) being pain free in last 24 hours of life; and (5) presence of family members or other significant persons at the time of death. Being delirium free was defined as having no episodes of delirium in the last 24 hours of life among patients with at least 1 delirium assessment; an episode of delirium was defined as a positive score on the Confusion Assessment Method for the ICU or a score greater than 4 on the Intensive Care Delirium Screening Checklist. Patients who were unconscious, as determined by a Richmond Agitation Sedation Scale score of −4 or −5, were not candidates for delirium assessment and, thus, were excluded from this measure. Being pain free was defined as having no significant pain episodes in last 24 hours of life among patients with at least 1 pain assessment; significant pain was defined by a numerical rating score greater than 3, a Critical Care Pain Observation Tool score greater than 2, or a Behavioral Pain Score greater than 3. Only communicative patients were eligible for numerical rating score assessment. Patients who were unconscious or otherwise unable to self-report were assessed using either the Critical Care Pain Observation Tool or Behavioral Pain Score, both validated scales based on behavioral manifestations of pain. Patients with missing data (ie, had no pain and/or no delirium assessments in the last 24 hours of life) were excluded from these measures. Data collection definitions from the standard operating procedure manual appear in eAppendix 1 in the Supplement.

### Statistical Analysis

We first summarized unit and patient characteristics, cohortwide adherence to structure and process measures, and overall EOL event rates. To evaluate unit-level variation, EOL event rates were stratified by unit and presented as medians, interquartile ranges (IQRs), and ranges. As a sensitivity analysis, we reevaluated unit-level variation and EOL event rates after excluding decedents who had been admitted in the precollaborative period. We conducted a second sensitivity analysis, designating all patients with missing data for EOL pain or delirium assessments as pain free or delirium free, respectively. Results of sensitivity analyses were consistent with our original analyses and are presented in eAppendix 2 in the Supplement.

We used generalized linear mixed models to explore the associations between the following variables and outcomes: (1) patient and unit characteristics with EOL events, (2) patient characteristics with patient-level process measures, and (3) process and structural measures with EOL events. Each EOL event was modeled as binomial with a logit link function and included a random effect for unit to account for association between patients within a unit. We used logistic regression to model associations between unit-level characteristics and unit-level structural measures.

To uncover unit-level patterns in EOL care without applying prespecified hypotheses about the distinct patterns of care delivery (ie, without defining a pattern of high-quality or low-quality performance), we conducted unsupervised clustering analysis using a k-means clustering algorithm with Euclidean distance. This technique identifies subgroups (ie, clusters of ICUs) by maximizing similarities within and differences between clusters according to selected features. Each unit’s rates for the 5 EOL events were input as features into the clustering algorithm; the optimal number of clusters was determined using the average silhouette method. We used Pearson χ^2^ test to determine whether the distribution of unit characteristics differed between clusters. A 2-tailed *P* < .05 defined statistical significance; analyses were conducted using R statistical software version 3.3.1 (R Project for Statistical Computing). Data were analyzed between August 2018 and June 2019.

## Results

The ILC included data collection for 16 945 adult patients with recorded age and discharge status; 1536 (9.1%) died during their initial ICU stay (Table 2).^34^ Of the 1536 decedents, 654 (42.6%) were women; 1037 (67.5%) were 60 years or older; and 1088 (70.8%) were identified as white individuals, 178 (11.6%) as black individuals, 65 (4.2%) as Asian individuals, and 197 (12.8%) as other, which included Hawaiian, American Indian, and patients identified as other race. The ILC comprised 68 units; 11 (16.2%) medical, 8 (11.8%) surgical, 43 (63.2%) mixed or other specialty units, and 6 (8.8%) with missing data for ICU type. Of the participating units, 42 (61.8%) were in teaching hospitals; 23 (33.8%) were located in the eastern United States, 22 (32.4%) in the Midwest, and 23 (33.8%) in the western United States. The median number of decedents per ICU was 20 (IQR, 11-31; range, 1-93). The distribution of decedents by ICU type was 332 decedents (21.6%) in medical units, 91 (5.9%) in surgical units, and 909 (59.2%) in mixed specialty and other units; 204 decedents (13.3%) had missing ICU type data.

**Table 2.** Characteristics of 1536 Decedents and 68 Units

Characteristic	No./Total No. (%)^a^
Decedent characteristic	
Women	654/1531 (42.7)
Age, y	
18-39	125/1536 (8.1)
40-59	374/1536 (24.4)
60-79	388/1536 (25.3)
≥80	649/1536 (42.2)
Race^b^	
White	1088/1536 (70.8)
Black	178/1536 (11.6)
Asian	65/1536 (4.2)
Other^c^	197/1536 (12.8)
Unknown	8/1536 (0.5)
Hispanic ethnicity	181/1482 (12.2)
ICU LOS, median (IQR), d	4.5 (2.5-8.0)
Hospital LOS, median (IQR), d	6.0 (3.0-10.5)
Primary admitting diagnosis^d^	
Sepsis or septic shock	511/1536 (33.3)
Pneumonia	221/1536 (14.4)
Renal failure	196/1536 (12.8)
Acute myocardial infarction or cardiogenic shock	143/1536 (9.3)
Change in mental status	125/1536 (8.1)
Malignant neoplasm	123/1536 (8.0)
Arrhythmia	122/1536 (7.9)
Metabolic, endocrine, or electrolyte	120/1536 (7.8)
Congestive heart failure	97/1536 (6.3)
COPD or asthma	88/1536 (5.7)
Unit characteristic	
ICU type	
Medical	11/62 (17.7)
Surgical^e^	8/62 (12.9)
Mixed specialty or other^f^	43/62 (69.4)
Hospital size, beds	
≤351	20/61 (32.8)
352-489	21/61 (34.4)
≥490	20/61 (32.8)
Teaching hospital	42/64 (65.6)
Hospital location	
Urban	38/64 (59.4)
Suburban	18/64 (28.1)
Rural	8/64 (12.5)
Geographic region	
East	23/68 (33.8)
Midwest	22/68 (32.3)
West	23/68 (33.7)
Funding structure	
Private	40/64 (62.5)
Public or federal	24/64 (37.5)
Palliative care services available	56/61 (91.8)
Admitting structure	
Closed	23/64 (35.9)
Open	21/64 (32.8)
Semiopen	20/64 (31.3)
Coverage in unit	
Intensivist^g^	61/63 (96.8)
Resident	37/61 (60.7)
Critical care fellow	21/63 (33.3)
Advanced practice provider	34/63 (54.0)

Abbreviations: COPD, chronic obstructive pulmonary disease; ICU, intensive care unit; IQR, interquartile range; LOS, length of stay.

^a^ Total number of patients or units for individual characteristics can be less than 1536 or 68, respectively, due to missing data. Statistics are calculated out of available patients and units.

^b^ Two patients were identified with 2 race categories.

^c^ Includes Hawaiian, American Indian, and patients identified as other race.

^d^ Frequency of 10 most frequent admitting diagnoses. Patients could be assigned more than 1 diagnosis as applicable.

^e^ Designates surgical, cardiothoracic, trauma, or burn ICUs.

^f^ Includes neurologic or neurosurgical ICUs.

^g^ Intensivist as defined by the Leapfrog Group.^34^

### Structures, Processes, and Outcomes of EOL Care

Table 3 displays adherence rates for structural and process measures and EOL event rates within the entire study sample. A total of 47 of 60 units (78.3%) had an open visitation policy for visitors, but only 18 of 60 (30.0%) had a structured opportunity for staff to reflect about the experience caring for patients who are dying. Of 1520 decedents, 1380 (90.8%) had at least 1 pain assessment in the last 24 hours of life, but only 913 of 1522 decedents (60.0%) had at least 1 delirium assessment during the same period. Patients experienced a median (IQR) of 3 (1-6) significant pain episodes in the last 24 hours of life. A total of 616 of 1527 patients (40.3%) had ascertainment of an AD during their hospitalization. Logistic regression models testing the associations of unit and patient characteristics with structural and process measures appear in eTable 1 and eTable 2 in the Supplement.

**Table 3.** Structures, Processes, and Outcomes of EOL ICU Care

Measure	No./ Total No. (%)
Structure: unit-level quality measures	
Policy for open visitation	47/60 (78.3)
EOL-specific protocols for general symptom management	44/62 (71.0)
EOL-specific protocols for withdrawal of mechanical ventilation	34/62 (54.8)
Policy for continuity of nursing services	31/60 (51.7)
Policy for structured clinician reflection opportunity	18/60 (30.0)
Process: patient-level quality measures	
Assessment of pain in the last 24 h of life	1380/1520 (90.8)
Offer or delivery of spiritual support during ICU stay	963/1506 (63.9)
Assessment of delirium in the last 24 h of life	913/1522 (60.0)
Ascertainment of advance directive during hospitalization	616/1527 (40.3)
Outcome: patient-level EOL events	
Absence of cardiopulmonary resuscitation in last hour of life	1348/1536 (87.8)
Family or significant persons present at time of death	1226/1536 (79.8)
Pain free in last 24 h of life^a^	999/1380 (72.4)
Extubated prior to death^b^	867/1350 (64.2)
Delirium free in the last 24 h of life^c^	538/913 (58.9)

Abbreviations: EOL, end of life; ICU, intensive care unit.

^a^ Among patients with a documented pain assessment.

^b^ Among patients receiving mechanical ventilation.

^c^ Among patients with a documented delirium assessment.

**Figure 1.**
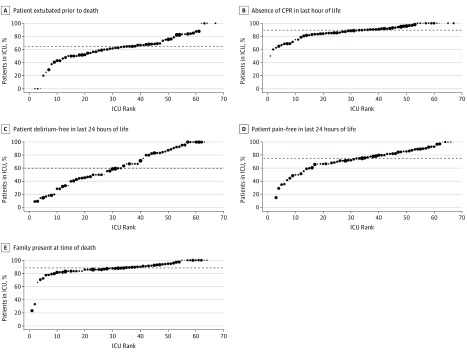
Variation in End-of-Life Events Among Intensive Care Units (ICUs) in the United States Each circle represents a single ICU, and the y-axis value represents the percentage of decedents in that unit who experienced the event. Units are ranked from 1, representing the lowest end-of-life event frequency, to 68, representing the highest event frequency, along the x-axis. The size of each circle is proportional to the total number of decedents in that unit. The dotted lines indicate the median event rate among all units. CPR indicates cardiopulmonary resuscitation.

Figure 1 demonstrates the wide unit-level variability in EOL event rates. Stratified by unit, the median event rates were as follows: extubation prior to death, 64.7% (IQR, 51.5%-77.3%; range, 0-100%); absence of CPR in last hour of life, 89.5% (IQR, 83.3%-96.1%; range, 50.0%-100%); delirium free in last 24 hours of life, 60.0% (IQR, 43.7%-85.2%; range, 9.1%-100%); pain free in last 24 hours of life, 75.1% (IQR, 66.0%-85.7%; range, 0-100%); and family present at time of death, 88.2% (IQR, 83.3%-94.7%; range 0-100%). Table 4 displays the multivariable adjusted models evaluating associations of patient and unit characteristics with each EOL event. Compared with white individuals, black individuals were associated with lower odds of absence of CPR in last hour of life (adjusted odds ratio [aOR], 0.33; 95% CI, 0.20-0.56; *P* < .001) and lower odds of extubation before death (aOR, 0.59; 95% CI, 0.39-0.90; *P* = .02). Black individuals were also associated with increased odds of being pain free before death (aOR, 1.92; 95% CI, 1.08-3.45; *P* = .03). Compared with ICUs in the eastern United States, those in the Midwest were associated with higher odds of absence of CPR in the last hour of life (aOR, 2.04; 95% CI, 1.06-3.85; *P* = .03) and of extubation before death (aOR, 2.23; 95% CI, 1.39-3.58; *P* < .001). Surgical ICUs were associated with higher odds of extubation before death (aOR, 2.47; 95% CI, 1.04-5.69; *P* = .03) and lower odds of being pain free (aOR, 0.19; 95% CI, 0.04-0.79; *P* = .02) compared with medical ICUs.

**Table 4.** Multivariable aORs of EOL Events for Patient and Hospital Characteristics

Characteristic	Extubated Before Death (n = 989)		Absence of CPR in Last Hour of Life (n = 989)		Delirium Free in Last 24 h of Life (n = 759)^a^		Pain Free in Last 24 h of Life (n = 1022)		Family Present at Time of Death (n = 1042)	
	aOR (95% CI)	*P* Value	aOR (95% CI)	*P* Value	aOR (95% CI)	*P* Value	aOR (95% CI)	*P* Value	aOR (95% CI)	*P* Value
Patient characteristic										
Sex										
Women	1 [Reference]	NA	1 [Reference]	NA	1 [Reference]	NA	1 [Reference]	NA	1 [Reference]	NA
Men	0.90 (0.68-1.19)	.46	0.73 (0.50-1.06)	.10	1.00 (0.70-1.41)	.99	1.06 (0.78-1.45)	.69	0.75 (0.51-1.09)	.13
Age, y										
18-39	1 [Reference]	NA	1 [Reference]	NA	1 [Reference]	NA	1 [Reference]	NA	1 [Reference]	NA
40-59	2.88 (1.70-4.88)	<.001	0.74 (0.33-1.67)	.47	0.68 (0.31-1.47)	.32	0.97 (0.50-1.92)	.93	0.25 (0.08-0.73)	.01
60-79	3.23 (1.90-5.50)	<.001	0.83 (0.36-1.89)	.65	0.60 (0.28-3.59)	.20	0.70 (036-1.35)	.29	0.37 (0.12-1.13)	.08
≥80	2.53 (1.53-4.18)	<.001	0.68 (0.31-1.49)	.33	0.52 (0.25-1.09)	.08	0.72 (0.38-1.37)	.32	0.25 (0.08-0.71)	.01
Race										
White	1 [Reference]	NA	1 [Reference]	NA	1 [Reference]	NA	1 [Reference]	NA	1 [Reference]	NA
Asian	0.64 (0.32-1.28)	.21	2.94 (0.82-11.1)	.10	0.95 (0.36-2.56)	.93	1.47 (0.67-3.33)	.33	1.8 (0.57-5.71)	.32
Black	0.59 (0.39-0.90)	.02	0.33 (0.20-0.56)	<.001	1.28 (0.72-2.27)	.40	1.92 (1.08-3.45)	.03	0.65 (0.37-1.14)	.14
Other	0.65 (0.39-1.08)	.10	0.94 (0.49-1.85)	.87	1.01 (0.53-1.96)	.97	1.25 (0.68-2.33)	.47	0.74 (0.37-1.46)	.38
Hispanic ethnicity	0.75 (0.45-1.26)	.28	0.85 (0.43-1.69)	.64	1.22 (0.51-2.94)	.66	1.43 (0.74-2.78)	.30	1.07 (0.49-2.34)	.87
ICU LOS, per additional SD	1.06 (0.88-1.29)	.52	0.89 (0.74-1.09)	.26	NA	NA	1.22 (0.89-1.67)	.21	1.07 (0.70-1.63)	.77
Hospital LOS, per additional SD	0.82 (0.68-0.99)	.04	1.16 (0.90-1.52)	.24	NA	NA	0.90 (0.69-1.18)	.44	1.03 (0.73-1.46)	.85
Hospital characteristic										
ICU type										
Medical	1 [Reference]	NA	1 [Reference]	NA	1 [Reference]	NA	1 [Reference]	NA	1 [Reference]	NA
Surgical^b^	2.47 (1.07-5.69)	.03	1.25 (0.37-4.17)	.72	NA	NA	0.19 (0.04-0.79)	.02	1.13 (0.25-5.09)	.87
Mixed specialty or other^c^	1.43 (0.88-2.33)	.14	1.41 (0.67-2.94)	.38	NA	NA	0.66 (0.28-1.59)	.36	1.12 (0.47-2.67)	.80
Hospital size, beds										
≤351	1 [Reference]	NA	1 [Reference]	NA	1 [Reference]	NA	1 [Reference]	NA	1 [Reference]	NA
352-489	1.28 (0.77-2.14)	.34	1.14 (0.56-2.33)	.72	NA	NA	0.97 (0.42-2.22)	.95	1.56 (0.67-3.6)	.30
≥490	2.54 (1.47-4.41)	<.001	2.27 (1.05-5.00)	.04	NA	NA	0.66 (0.27-1.64)	.36	1.14 (0.48-2.73)	.77
Teaching hospital	1.09 (0.41-2.92)	.86	1.72 (0.38-7.69)	.48	NA	NA	1.32 (0.26-6.67)	.74	2.25 (0.41-12.38)	.35
Hospital location										
Urban	1 [Reference]	NA	1 [Reference]	NA	1 [Reference]	NA	1 [Reference]	NA	1 [Reference]	NA
Suburban	1.55 (0.91-2.64)	.10	1.15 (0.56-2.38)	.70	0.53 (0.22-1.25)	.15	1.45 (0.67-3.13)	.35	1.1 (0.5-2.44)	.81
Rural	1.89 (0.96-3.69)	.06	1.79 (0.67-4.76)	.25	0.89 (0.26-3.03)	.86	1.56 (0.53-4.55)	.42	1.89 (0.63-5.67)	.26
Geographic region										
East	1 [Reference]	NA	1 [Reference]	NA	1 [Reference]	NA	1 [Reference]	NA	1 [Reference]	NA
Midwest	2.23 (1.39-3.58)	<.001	2.04 (1.06-3.85)	.03	NA	NA	0.97 (0.47-2.04)	.95	1.02 (0.5-2.11)	.95
West	1.04 (0.65-1.66)	.87	1.27 (0.65-2.44)	.49	NA	NA	0.70 (0.33-1.45)	.34	0.88 (0.41-1.88)	.74
Funding structure										
Private	1 [Reference]	NA	1 [Reference]	NA	1 [Reference]	NA	1 [Reference]	NA	1 [Reference]	NA
Public/federal funding	1.79 (1.15-2.78)	.01	1.10 (0.57-2.08)	.79	NA	NA	0.71 (0.35-1.45)	.35	1.2 (0.58-2.45)	.63
Palliative care services available	1.57 (0.69-3.57)	.28	1.25 (0.43-3.57)	.68	NA	NA	0.77 (0.22-2.63)	.68	2.65 (0.78-8.96)	.12
Admitting structure										
Open	1 [Reference]	NA	1 [Reference]	NA	1 [Reference]	NA	1 [Reference]	NA	1 [Reference]	NA
Semiopen	0.62 (0.4-0.95)	.03	0.73 (0.38-1.39)	.33	NA	NA	0.89 (0.45-1.79)	.75	1.05 (0.53-2.09)	.88
Closed	1.18 (0.70-2.00)	.54	0.74 (0.34-1.64)	.45	NA	NA	0.82 (0.34-2.00)	.66	0.91 (0.37-2.25)	.85
Coverage in unit										
Intensivist^d^	2.01 (0.56-7.29)	.29	1.01 (0.15-6.67)	.99	8.33 (0.80-100)	.08	1.37 (0.24-7.69)	.72	0.52 (0.05-5.96)	.60
Resident	0.94 (0.33-2.70)	.91	0.71 (0.15-3.33)	.66	NA	NA	0.68 (0.13-3.57)	.65	0.53 (0.09-3.19)	.49
Critical care fellow	0.78 (0.46-1.32)	.36	0.95 (0.43-2.08)	.91	NA	NA	1.15 (0.45-2.94)	.77	1.40 (0.55-3.61)	.48
Advance practice provider	0.43 (0.25-0.74)	.002	0.90 (0.42-1.92)	.79	1.92 (0.89-4.17)	.10	1.61 (0.74-3.57)	.23	0.76 (0.35-1.67)	.50

Abbreviations: aOR, adjusted odds ratio; CPR, cardiopulmonary resuscitation; EOL, end of life; ICU, intensive care unit; LOS, length of stay; NA, not applicable.

^a^ The exploratory delirium model including all patient and hospital characteristics failed to converge; the model did not converge after restarting from prior fits and applying alternate optimizers. Thus, only independent variables with significant association (ie, *P* < .05) with delirium on univariate logistic regression analysis were included in the final model.

^b^ Designates surgical, cardiothoracic, trauma, or burn ICUs.

^c^ Includes neurologic or neurosurgical ICUs.

^d^ Intensivist as defined by the Leapfrog Group.^34^

### Patterns of EOL Care Delivery

**Figure 2.**
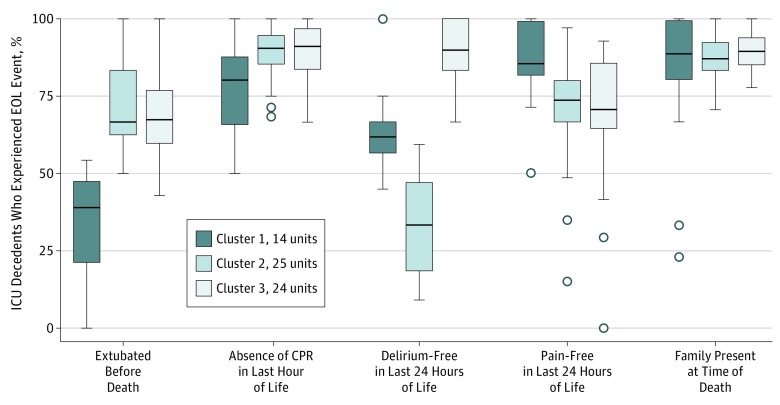
Unit-Level Patterns of End-of-Life (EOL) Care Delivery Cluster analysis revealed 3 mutually exclusive, unit-level patterns of end-of-life care delivery. Of 63 intensive care units (ICUs) in this analysis, 14 (22.2%) belonged to cluster 1, which had the lowest rate of extubation before death and the lowest rate of cardiopulmonary resuscitation (CPR) avoidance but the highest pain-free rate. The 25 units (39.7%) belonging to cluster 2 had the lowest delirium-free rate but high rates of all other EOL events. The 24 units (38.1%) belonging to cluster 3 had consistently high rates across all 5 EOL events. The upper and lower bounds of the boxes represent the 75th and 25th percentiles, respectively. The midbox horizontal line represents the median. The maximum and minimum observations are indicated by the vertical whiskers, and outliers are indicated by circles.

Unsupervised cluster analysis revealed 3 mutually exclusive unit-level patterns of EOL care delivery (Figure 2; eFigure 1 and eFigure 2 in the Supplement). We excluded 5 units from the analysis because of missing data for the delirium EOL event. The 14 units (22.2%) belonging to cluster 1 had the lowest rate of extubation before death and the lowest rate of CPR avoidance but achieved the highest pain-free rate. The 25 units (39.7%) belonging to cluster 2 had the lowest delirium-free rate but achieved high rates of all other EOL events. The 24 units (38.1%) belonging to cluster 3 achieved consistently high rates across all 5 EOL events. Unit characteristics stratified by cluster and pairwise comparisons of event rates between clusters appear in eTable 3 and eTable 4 in the Supplement. Among all measured unit characteristics, the only significant association with cluster membership was advance practice provider coverage in the unit (cluster 1, 66.7% of units with advance practice providers; cluster 2, 30.4%; cluster 3, 73.9%; *P* = .008).

### Association Between Structural, Process, and Outcomes Measures

At the patient level, documented ascertainment of an AD was associated with lower odds of receiving CPR in the last hour of life (OR, 0.70; 95% CI, 0.49-0.99; *P* = .04), and a documented offer or delivery of spiritual support was associated with higher odds of having family present at the time of death (OR, 1.95; 95% CI, 1.37-2.77; *P* < .001). Patients who died in a unit with an open visitation policy were more likely to experience pain in the last 24 hours of life (OR, 2.21; 95% CI, 1.15-4.27; *P* = .02). The full results of univariate mixed-effects logistic regression models testing associations between structure and process measures and EOL events appear in eTable 5 and eTable 6 in the Supplement. Having unit protocols for EOL symptom management was not associated with the presence of delirium or pain in the last 24 hours of life (eTable 6 in the Supplement).

## Discussion

In this cohort of 1536 decedents from a diverse sample of 68 ICUs across the United States, we found wide unit-level variation in the delivery of EOL care for patients who died in an ICU. We demonstrated that unit-level variation in EOL care extended beyond the delivery of CPR and mechanical ventilation near death and substantially affected symptom control at the EOL, including delirium. We also uncovered 3 unit-level patterns of care delivery that suggested meaningful differences in the experience of death and dying for patients in higher-performing and lower-performing units. To achieve optimal care for all patients who die in an ICU, future research should target unit-level variation and investigate and disseminate the successes of high-performing units.

### Outcomes of EOL ICU Care

Minimizing pain and other burdensome symptoms, such as delirium, near death is a near-universal goal for patients and their loved ones.^35,36^ However, important trade-offs in symptom control may be necessary and depend on patient values, goals, and preferences; thus, the optimal rate of pain or delirium at EOL is unknown, and our study did not address whether EOL events were aligned with patient-identified or family-identified EOL priorities. Overall, approximately 1 in 4 patients in this study experienced at least 1 significant pain episode in the last day of life, which is similar to the rate of EOL pain in recent studies of hospitalized decedents not limited to the ICU setting.^37,38^ However, the stark differences between units, which ranged from no patients to almost all patients with at least 1 episode of EOL pain or delirium, suggest unwarranted inconsistency in the delivery of care. In a multivariable model, we found that dying in a surgical ICU was the only patient or unit characteristic significantly associated with the presence of EOL pain. More than 70% of units in this study had protocols for EOL symptom management. However, we found no association between the presence of EOL symptom protocols and EOL symptoms for individual patients. A more granular understanding of the contents of EOL symptom management protocols, how the protocols are implemented at the bedside, and whether these protocols lead to optimal EOL symptom management is a critical next step to improve EOL ICU care.

This study provided a national estimate of the prevalence of EOL ICU delirium, a previously underappreciated symptom for patients who die in an ICU. While the impact of delirium in the general ICU population is well established,^39^ little is known about the distinctive features of EOL ICU delirium, including assessment challenges, management considerations, and the consequences for patients, families, and clinicians. We speculate that EOL ICU delirium is uniquely important for patients and families, given the strong desire for patients and families to have meaningful interactions with family and friends near the EOL.^36^ Among decedents in this study who were assessed for delirium, more than 40% experienced delirium in the last day of life, similar to rates described in inpatient hospice and palliative care units.^40,41^ In the multivariable model, we found no significant associations of patient or unit characteristics with the presence of EOL delirium. Given the favorable staffing ratios and highest available level of care in an ICU, we believe ICUs can be expected to perform even better than other care venues in managing EOL delirium. However, notable challenges to this aspiration exist, given the risk factors for EOL delirium related to critical illness and the ICU environment. Another 40% of decedents in ICUs were never assessed for delirium in the last day of life, despite the favorable staffing ratios in ICUs and the prevalence and burdens of this syndrome. This finding may be partially explained by the number of patients who were unconscious near EOL (ie, Richmond Agitation-Sedation Scale score, −4 or −5) and, thus, not candidates for delirium assessment by the 2 scales used in the ILC. Moreover, this study was conducted within a quality improvement collaborative designed to improve the assessment and management of delirium, and thus, our findings may overestimate delirium assessment and underestimate the prevalence of uncontrolled EOL ICU delirium in the United States.

Our cluster analysis identified 3 previously unrecognized unit-level patterns in the delivery of EOL care. These patterns suggest meaningful differences in the EOL experience for patients and their families in higher-performing and lower-performing units. These patterns were not associated with commonly recognized unit characteristics, such as admitting structure, ICU type, geographic location, or teaching status. This finding suggests latent features of a unit, such as local culture and ethical climate, may have an important influence on EOL care delivery. Approximately 1 in 3 units in this study achieved high performance across all measured domains of EOL care. Units belonging to this cluster should be further studied using qualitative methods to understand the specific mechanisms that promote this high-quality EOL care.

A second cluster achieved similarly high performance, with the exception of higher rates of EOL ICU delirium. This finding further supports our conclusion that EOL ICU delirium warrants further attention to understand whether the stark difference between clusters 2 and 3 can be explained by detection, reporting, or management. Finally, the smallest cluster, representing 22% of units, was characterized by high rates of invasive therapies until the time of death. Units in this cluster had a low rate of extubation before death compared with other units, and the rate of CPR in the last hour of life was double the rate in other clusters. This cluster simultaneously had the highest rate of pain-free patients. This hypothesis-generating finding may reflect sedation and analgesia practices for patients who are intubated vs extubated and deserves close attention through future studies that explicitly acknowledge and evaluate the necessary trade-offs in EOL care to ultimately improve the complex process of delivering goal-concordant EOL ICU care.^42^

### Processes of EOL ICU Care

This study identified several additional gaps in the processes of EOL care delivery. The presence or absence of an AD was documented for only 40% of decedents in an ICU during hospitalization. This finding likely reflects, in part, poor overall uptake of ADs given that only one-third of adults in the United States have completed AD documentation.^43^ However, ADs remain the most widely adopted tool in advance care planning and have the potential to support the alignment of EOL care with patient preferences.^44^ Moreover, this process measure evaluated whether any clinician documented whether a patient had an AD (including the status of no AD), suggesting that our findings also reflect poor attention to ADs among clinicians. Individuals who die in an ICU are the precise population to whom ADs apply, and so the low rate of AD documentation in this study suggests a continued need to increase advance care planning efforts before a critical illness and to improve ascertainment of ADs during a critical illness. We also found that 40% of decedents in the ICU failed to receive any offer of spiritual support during their ICU stay. Clinicians in the ICU generally believe it is their responsibility to address religious and spiritual needs of patients,^45^ and insufficient spiritual support is associated with increased symptoms of posttraumatic stress disorder for surrogates of patients in the ICU.^46^ Thus, efforts are needed to increase the provision of spiritual support for patients who die in the ICU and their families.

### Structures of EOL ICU Care

The vast majority of units in this study allowed open visitation, which conflicts with a study published in 2013 that found restricted visitation hours in 80.4% of surveyed ICUs in the United States.^47^ This discrepancy may be associated with increased attention during the last decade on fostering patient-centered and family-centered ICU environments, or it may reflect a latent characteristic of the units that chose to participate in the ILC.^48,49^ In exploratory analyses, we also found an unanticipated association; patients who died in units with open visitation policies were more likely to experience pain at the EOL. Patients with visitors may make deliberate trade-offs between meaningful interaction and pain control with sedating medications. Alternatively, visitors at the bedside may be more likely to call attention to patients’ pain symptoms. This hypothesis-generating finding and potentially unintended consequence of open visitation warrants further study. Less than one-third of units in this study provided structured opportunities for clinicians to reflect on caring for dying patients. Given increased awareness about the impact of providing EOL care on professional burnout in the ICU,^50,51^ structured, unit-level support for clinicians who care for dying patients may provide a feasible strategy to mitigate this growing problem.^52,53^

### Limitations

This study has limitations. This large cohort of decedents in the ICU were cared for in a diverse sample of units from across the United States. However, the culture and other latent characteristics of units that electively chose to participate in the ILC may differ from nonparticipating units in an unpredictable manner, which may limit the generalizability of our findings. The ILC was focused on pain and delirium; thus, our findings may overestimate the performance of pain and delirium assessment and management at the EOL across the United States. Patient-level data collection within the ILC was also limited to the first 15 consecutive patients admitted per month and excluded patients who died or underwent withdrawal of life-sustaining treatments within the first 24 hours of ICU admission, which may have led to a biased or nonrepresentative sample of decedents. We were unable to conduct case-mix adjustment by severity of illness because of a lack of uniform severity of illness reporting. Participating units were responsible for data collection and received formal data collection training and a detailed standard operating procedures manual with data definitions. However, it is possible that variation and errors may have occurred, introducing potential reporting bias. Our findings only represent quality measures and events that could be feasibly collected through the ILC. The proposed EOL quality measures evaluated in this study are supported by bioethical principles and limited observational data,^2,7,8^ but whether these measures truly represent the most important features of EOL care remains unknown. For example, process measures that rely on clinician documentation do not fully represent care delivered. Most importantly, these measures do not reflect whether observed EOL events are aligned with individual patients’ values, goals, or preferences. Furthermore, we selected a flat clustering method to explore patterns of EOL care delivery, which produced a single partitioning of the data. This enhanced the interpretability of our findings, but alternate clustering methods may have yielded slightly different results.

## Conclusions

This study suggests most decedents in the ICU avoid CPR at EOL, have family present at their bedside, and are closely assessed for pain. However, the delivery of EOL care varies widely among units in the United States, including the rate of pain and delirium near death. Unit-level patterns of care delivery suggest meaningful differences in the EOL experiences of patients who die in higher-performing and lower-performing units. To achieve the best possible care for patients who die in an ICU, future research should target unit-level variation and investigate and disseminate the successes of high-performing units.

## Notes

Supplement. **eAppendix 1. **Measure Definitions **eAppendix 2. **Sensitivity Analyses **eFigure 1. **Cluster Plots for Results of K-Means Clustering Using 2, 3, 4, or 5 Clusters **eFigure 2. **Determination of the Optimal Number of Clusters Using the Average Silhouette Method **eTable 1. **Univariate Logistic Regression Models of Association Between Unit Characteristics and Unit-Level Structural Quality Measures **eTable 2. **Univariate Mixed Effects Logistic Regression Models of Association Between Patient Characteristics and Patient-Level Process Quality Measures **eTable 3. **Comparison of Unit Characteristics Across Clusters **eTable 4. **Pairwise Comparisons of End-of-Life Event Rates Across Clusters **eTable 5. **Univariate Mixed Effects Logistic Regression Models of Association Between Patient-Level Process Quality Measures and EOL Events **eTable 6. **Univariate Mixed Effects Logistic Regression Models of Association Between Unit-Level Structural Quality Measures and End-of-Life Events
