# Supplementary material for: Assessment of Variability in End-of-Life Care Delivery in Intensive Care Units in the United States
Source: JAMA Netw Open. 2019 Dec 11;2(12):e1917344. doi: 10.1001/jamanetworkopen.2019.17344 (PMC6991207; doi:10.1001/jamanetworkopen.2019.17344)
Supplement: Supplement. — eAppendix 1. Measure Definitions eAppendix 2. Sensitivity Analyses eFigure 1. Cluster Plots for Results of K-Means Clustering Using 2, 3, 4, or 5 Clusters eFigure 2. Determination of the Optimal Number of Clusters Using the Average Silhouette Method eTable 1. Univariate Logistic Regression Models of Association Between Unit Characteristics and Unit-Level Structural Quality Measures eTable 2. Univariate Mixed Effects Logistic Regression Models of Association Between Patient Characteristics and Patient-Level Process Quality Measures eTable 3. Comparison of Unit Characteristics Across Clusters eTable 4. Pairwise Comparisons of End-of-Life Event Rates Across Clusters eTable 5. Univariate Mixed Effects Logistic Regression Models of Association Between Patient-Level Process Quality Measures and EOL Events eTable 6. Univariate Mixed Effects Logistic Regression Models of Association Between Unit-Level Structural Quality Measures and End-of-Life Events [file jamanetwopen-2-e1917344-s001.pdf]

# Supplementary Online Content

Kruser JM, Aaby DA, Stevenson DG, et al. Assessment of variability in end-of-life care delivery in intensive care units in the United States. *JAMA Netw Open*. 2019;2(12):e1917344. doi:10.1001/jamanetworkopen.2019.17344

**eAppendix 1.** Measure Definitions
**eAppendix 2.** Sensitivity Analyses
**eFigure 1.** Cluster Plots for Results of K-Means Clustering Using 2, 3, 4, or 5 Clusters
**eFigure 2.** Determination of the Optimal Number of Clusters Using the Average Silhouette Method
**eTable 1.** Univariate Logistic Regression Models of Association Between Unit Characteristics and Unit-Level Structural Quality Measures
**eTable 2.** Univariate Mixed Effects Logistic Regression Models of Association Between Patient Characteristics and Patient-Level Process Quality Measures
**eTable 3.** Comparison of Unit Characteristics Across Clusters
**eTable 4.** Pairwise Comparisons of End-of-Life Event Rates Across Clusters
**eTable 5.** Univariate Mixed Effects Logistic Regression Models of Association Between Patient-Level Process Quality Measures and EOL Events
**eTable 6.** Univariate Mixed Effects Logistic Regression Models of Association Between Unit-Level Structural Quality Measures and End-of-Life Events

This supplementary material has been provided by the authors to give readers additional information about their work.

© Kruser JM et al. *JAMA Network Open.*

**eAppendix 1.** Measure Definitions
**End-of-life care measure entries from the Standard Operating Procedure Manual:**

A. *Documentation of Advance Directive*: Check “Yes” if there is documentation of the presence or absence of an advance directive in the medical record at any point in the patient’s hospitalization or ICU stay. An advance directive is a legal document in which a patient specifies actions to be taken or specifies a person to make healthcare decisions on the patient’s behalf if the patient is unable to make his/her own healthcare decisions. Examples of advance directives include healthcare power of attorney, healthcare proxy, and living will. This does not include a “do-not-resuscitate” order. This is often documented as part of the admission process at many hospitals. Data entry options: (1) Yes; (2) No.

B. *Documentation of Spiritual Support during the ICU Collaborative Stay*: Check “Yes” if there is documentation of an offer of spiritual support to the patient or to family members during the pilot ICU stay. An offer of spiritual support includes any offer from any member of the healthcare team to the patient or to family members that involves spiritual care services, chaplain services, or a pastoral care visit. This includes hospital-provided services, such as a visit from the hospital chaplain, or spiritual services from an outside clergy member or other spiritual advisor, including practices such as last rites. This information may be found in progress or consultation notes written by hospital chaplains, orders for spiritual support/chaplain consultation, or in nursing or physician documentation. Data entry options: (1) Yes; (2) No.

C. *Was delirium present any time during the 24 hours before death*? Check “Yes” if there is documentation of delirium during this period. Delirium presence is defined as documentation of a patient having a Confusion Assessment Method for the ICU (CAMICU) score that is positive or an Intensive Care Delirium Screening Checklist (ICDSC) > 4. If the patient was not delirious during this period, using the CAM-ICU and/or ICDSC tools, check “No.” Data entry options: (1) Yes, documented present; (2) No, documented absent; (3) No Assessment results documented.

D. *Number of documented significant pain episodes during the 24 hours before death*. Record only the number of significant pain episodes that were documented during the 24 hours before death, using the Numerical Rating Scale (NRS), Behavioral Pain Scale (BPS), and/or Critical-Care Pain Observation Tool (CPOT) pain assessment tools. Check all the scales that had documented NRS significant pain assessments defined as NRS > 3, CPOT > 2 and BPS >3 in the 24 hours prior to death. Check which tool was used and enter the corresponding number of significant pain episodes. Significant pain episodes are defined as any episode of pain indicated by an NRS score > 3, CPOT score > 2, and or BPS score > 3. Count the number of significant pain episodes that occurred during the 24 hours before death. You may enter results for more than one tool. If no pain score using these tools was documented during the 24 hours before death, check the corresponding box. Data entry options: Question 1: (1) Yes, Significant pain documented; (2) No, Pain assessments documented but none were significant; (3) No, No pain assessments documented. Question 2: Number of significant pain assessments (text, Min: 0, Max: 24).

E. *Did the patient receive cardiopulmonary resuscitation (CPR) during the last hour of life*? Check “Yes” if the patient received CPR during the hour preceding death. CPR is defined as a medical procedure involving repeated compression of a patient’s chest and/or defibrillation performed in an attempt to restore the blood circulation and breathing of a person who has had a cardiac arrest. If the patient did not receive CPR during the last hour of life, check “No.” Data entry options: (1) Yes; (2) No.

F. *If the patient received mechanical ventilation in the ICU, was it discontinued before death*? Using the definition of mechanical ventilation described previously, check “Yes” if mechanical ventilation was discontinued and/or the patient was extubated before death (i.e., the endotracheal tube was removed). For patients with tracheostomies, check “Yes” if mechanical ventilation was discontinued before death. Check “No” if the patient was receiving mechanical ventilation at the time of death. Check “Never mechanically ventilated” if the patient had never been mechanically ventilated during the ICU stay. This information may be found in respiratory therapist documentation and orders, extubation orders,

nursing documentation, or declaration of death notes. Data entry options: (1) Yes; (2) No; (3) Never Mechanically Ventilated.

G. *Were family/friends present at the time of death*? Check "Yes" if there was documentation of any family member, friend or person with any significant relationship present with the patient at the time of death. This may be found in the nursing documentation at the time of death, the physician progress notes near the time of death, or in the declaration-of-death note. Data entry options: (1) Yes; (2) No; (3) Not documented.

© Kruser JM et al. *JAMA Network Open.*

**eAppendix 2. Sensitivity Analyses**

1. Exclusion of pre-collaborative patients.
    a. Objective: To determine whether our findings were impacted by including ICU decedents from prior to the initiation of the collaborative ("pre-collaborative patients"), we conducted a sensitivity analysis by excluding all pre-collaborative decedents.

    b. Analyses and Interpretation:

        i. We repeated the main summary statistics within the sensitivity analysis cohort, describing overall performance of patient-level process and structure measures. We found no significant differences between the overall cohort and the sensitivity analysis cohort for any measure, using difference of proportions tests:

| | **Number of patients meeting measure / Total number of patients (%)** | | |
|---|---|---|---|
| **Process: Patient-Level Quality Measures** | **Original Cohort** | **Sensitivity Analysis Cohort** | **p value** |
| Assessment of pain in the last 24 hours of life | 1,380 / 1,520 (91) | 1,233 / 1,339 (92) | 0.24 |
| Offer or delivery of spiritual support during ICU stay | 963 / 1,506 (64) | 861 / 1,327 (65) | 0.63 |
| Assessment of delirium in the last 24 hours of life | 913 / 1,522 (60) | 830 / 1,339 (62) | 0.29 |
| Ascertainment of advance directive during hospitalization | 616 / 1,527 (40) | 544 / 1,344 (40) | 0.97 |
| **Outcome: Patient-Level EOL Events** | | | |
| Absence of cardiopulmonary resuscitation in last hour of life | 1,348 / 1,536 (88) | 1,187 / 1,352 (88) | 1.00 |
| Family or significant person(s) present at time of death | 1,226 / 1,536 (80) | 1,081 / 1352 (80) | 0.96 |
| Pain-free in last 24 hours of life | 999 / 1,380 (72) | 886 / 1,233 (72) | 0.80 |
| Extubated prior to death | 867 / 1,350 (64) | 886 / 1,233 (64) | 0.91 |
| Delirium-free in the last 24 hours of life | 538 / 913 (59) | 485 / 830 (58) | 0.87 |

© Kruser JM et al. *JAMA Network Open.*

ii. We repeated the unit-stratified analyses describing the median, interquartile range, and overall range of end-of-life events in the sensitivity analysis cohort and found no major differences compared to our original findings that would impact our overall interpretation of wide unit-level variation:

| | **Original Cohort** Median [IQR, full range] | **Sensitivity Analysis Cohort** Median [IQR, full range] |
|---|---|---|
| **Extubated prior to death** | 0.65 [0.52—0.77, 0.00—1.00] | 0.62 [0.53—0.78, 0.00—1.00] |
| **Absence of CPR in last hour of life** | 0.89 [0.83—0.96, 0.50—1.00] | 0.89 [0.83—0.96, 0.33—1.00] |
| **Pain-free in last 24 hours of life** | 0.75 [0.66—0.86, 0.00—1.00] | 0.76 [0.60—0.86, 0.00—1.00] |
| **Delirium-free in last 24 hours of life** | 0.60 [0.44—0.85, 0.09—1.00] | 0.61 [0.36—0.83, 0.09—1.00] |
| **Family present at time of death** | 0.88 [0.83—0.95, 0.23—1.00] | 0.89 [0.83—0.97, 0.21—1.00] |

2. Alternate approach to missing data for pain-free and delirium-free end-of-life events.
   a. Objective: In our original analyses, our approach to patients with completely missing data for pain or delirium assessments in the last 24 hours of life was to exclude those patients from eligibility for the measure. To determine whether unit-level variation in pain and delirium assessment (i.e., data collection) had an important influence on our finding of unit-level variation, we conducted a sensitivity analysis with an alternate approach to missing data. In the sensitivity analysis, we coded any patient with no assessments (for either pain or delirium) as pain-free or delirium-free, respectively.

   b. Analysis and Interpretation: We repeated the unit-stratified analyses describing the median, interquartile range, and overall range of end-of-life events in the sensitivity analysis cohort and found no major differences compared to our original findings that would impact our major overall interpretation of wide unit-level variation. While the median, unit-stratified rate of delirium-free patients increased in the sensitivity analysis, the IQR and overall range remained similarly wide compared to the original cohort:

| | **Original Cohort** Median [IQR, full range] | **Sensitivity Analysis Cohort** Median [IQR, full range] |
|---|---|---|
| **Pain-free in last 24 hours of life** | 0.75 [0.66—0.86, 0.00—1.00] | 0.78 [0.67—0.89, 0.00—1.00] |
| **Delirium-free in last 24 hours of life** | 0.60 [0.44—0.85, 0.09—1.00] | 0.83 [0.57—0.95, 0.14—1.00] |

© Kruser JM et al. *JAMA Network Open.*

**eFigure 1. Cluster Plots for Results of K-Means Clustering Using 2, 3, 4, or 5 Clusters**

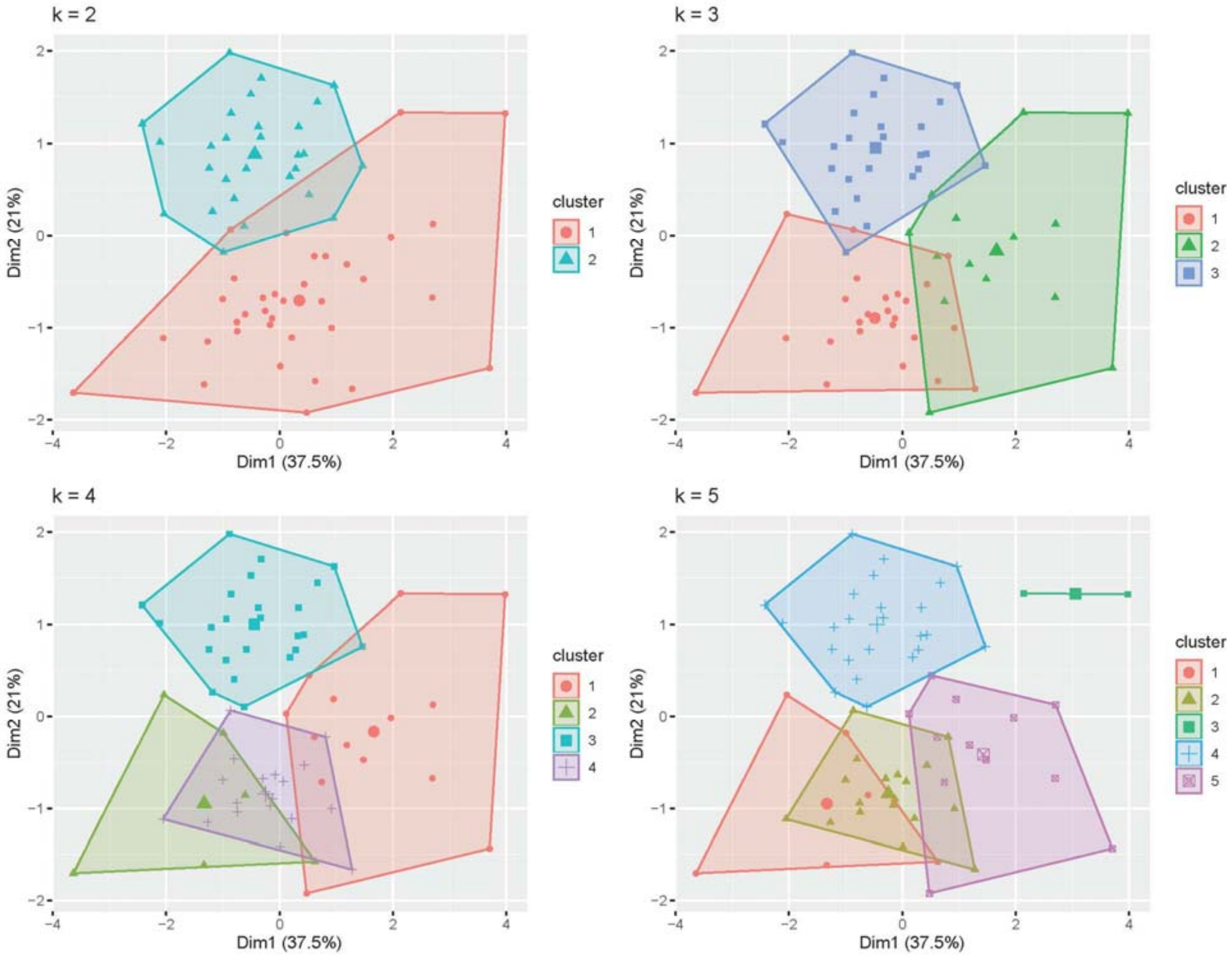


© Kruser JM et al. *JAMA Network Open.*

**eFigure 2. Determination of the Optimal Number of Clusters Using the Average Silhouette Method**

The findings suggest the optimal number of clusters is 3 (k = 3).

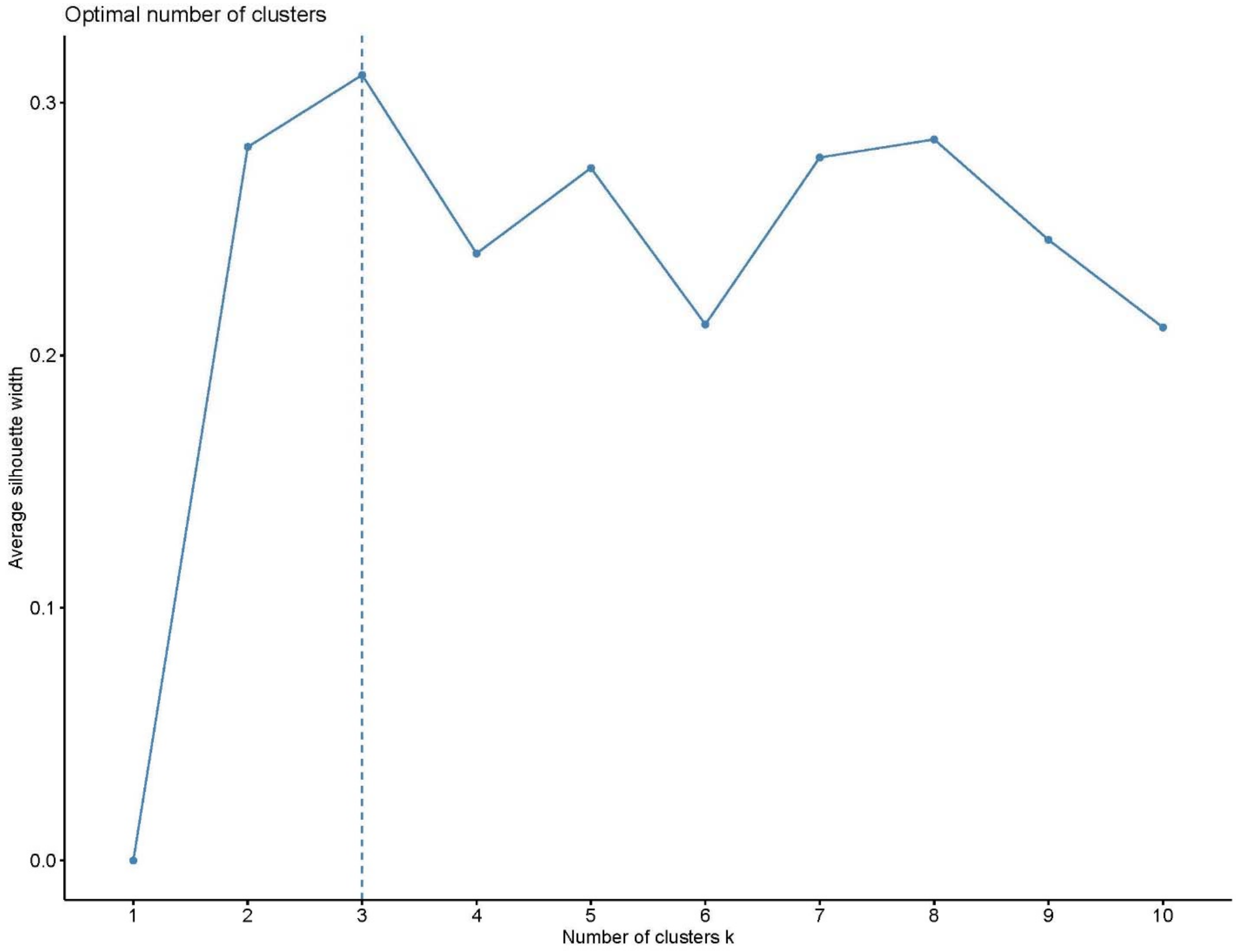


© Kruser JM et al. *JAMA Network Open.*

**eTable 1. Univariate Logistic Regression Models of Association Between Unit Characteristics and Unit-Level Structural Quality Measures (N=68)**

| Unit-Level Structural Quality Measure | Unit Characteristic (Independent Variables) | OR | 95% CI | | P value |
|---|---|---|---|---|---|
| Policy for Open Visitation | Hospital Region | | | | |
| | East | Reference | -- | -- | -- |
| | Midwest | 0.75 | 0.14 | 3.90 | 0.73 |
| | West | 0.56 | 0.11 | 2.77 | 0.48 |
| | ICU Location | | | | |
| | Urban | | | | |
| | Suburban | 0.56 | 0.13 | 2.43 | 0.44 |
| | Rural | 0.29 | 0.05 | 1.60 | 0.15 |
| | ICU Type | | | | |
| | Medical | Reference | -- | -- | -- |
| | Surgical | 0.13 | 8.98 | 0.95 | 0.13 |
| | Mixed/Other | 0.42 | 9.80 | 0.38 | 0.42 |
| | Teaching Hospital | 1.52 | 0.41 | 5.60 | 0.53 |
| | Hospital Size | | | | |
| | Small | Reference | -- | -- | -- |
| | Medium | 0.64 | 0.13 | 3.16 | 0.58 |
| | Large | 0.75 | 0.14 | 3.94 | 0.73 |
| End-of-life specific protocol(s) for general symptom management | Hospital Region | | | | |
| | East | Reference | -- | -- | -- |
| | Midwest | 1.65 | 0.48 | 5.69 | 0.43 |
| | West | 1.47 | 0.43 | 4.95 | 0.54 |
| | ICU Location | | | | |
| | Urban | Reference | -- | -- | -- |
| | Suburban | 0.34 | 0.10 | 1.10 | 0.07 |
| | Rural | 0.16 | 0.03 | 0.91 | 0.04 |
| | ICU Type | | | | |
| | Medical | Reference | -- | -- | -- |
| | Surgical | 1.39 | 0.22 | 8.92 | 0.73 |
| | Mixed/Other | 1.06 | 0.28 | 4.06 | 0.93 |
| | Teaching Hospital | 1.55 | 0.54 | 4.47 | 0.41 |
| | Hospital Size | | | | |
| | Small | Reference | -- | -- | -- |
| | Medium | 5.44 | 1.41 | 21.05 | 0.01 |
| | Large | 4.33 | 1.15 | 16.32 | 0.03 |
| End-of-life specific protocol(s) for withdrawal of mechanical ventilation | Hospital Region | | | | |
| | East | Reference | -- | -- | -- |
| | Midwest | 2.25 | 0.59 | 8.52 | 0.23 |
| | West | 3.19 | 0.79 | 12.80 | 0.10 |
| | ICU Location | | | | |
| | Urban | Reference | -- | -- | -- |
| | Suburban | 0.66 | 0.18 | 2.44 | 0.54 |
| | Rural | 0.17 | 0.03 | 0.85 | 0.03 |
| | ICU Type | | | | |
| | Medical | Reference | -- | -- | -- |
| | Surgical | 1.12 | 0.14 | 8.99 | 0.91 |
| | Mixed/Other | 0.91 | 0.20 | 4.01 | 0.90 |
| | Teaching Hospital | 2.67 | 0.86 | 8.32 | 0.09 |

© Kruser JM et al. *JAMA Network Open.*

| eTable 1 continued | | | | | |
|---|---|---|---|---|---|
| **Unit-Level Structural Quality Measure** | **Unit Characteristic (Independent Variables)** | **OR** | **95% CI** | | **P value** |
| End-of-life specific protocol(s) for withdrawal of mechanical ventilation | Hospital Size | | | | |
| | Small | Reference | -- | -- | -- |
| | Medium | 5.67 | 1.25 | 25.61 | 0.02 |
| | Large | 4.00 | 0.98 | 16.27 | 0.05 |
| Policy for continuity of nursing services | Hospital Region | | | | |
| | East | Reference | -- | -- | -- |
| | Midwest | 2.57 | 0.71 | 9.36 | 0.15 |
| | West | 2.57 | 0.71 | 9.36 | 0.15 |
| | ICU Location | | | | |
| | Urban | Reference | -- | -- | -- |
| | Suburban | 0.38 | 0.12 | 1.26 | 0.11 |
| | Rural | 0.18 | 0.03 | 1.04 | 0.06 |
| | ICU Type | | | | |
| | Medical | Reference | -- | -- | -- |
| | Surgical | 0.89 | 0.13 | 6.31 | 0.91 |
| | Mixed/Other | 0.67 | 0.16 | 2.73 | 0.57 |
| | Teaching Hospital | 9.00 | 2.48 | 32.68 | < 0.01 |
| | Hospital Size | | | | |
| | Small | Reference | -- | -- | -- |
| | Medium | 2.55 | 0.70 | 9.31 | 0.16 |
| | Large | 1.75 | 0.47 | 6.45 | 0.40 |
| Policy for structured clinician reflection opportunity | Hospital Region | | | | |
| | East | Reference | -- | -- | -- |
| | Midwest | 0.93 | 0.24 | 3.62 | 0.91 |
| | West | 0.93 | 0.24 | 3.62 | 0.91 |
| | ICU Location | | | | |
| | Urban | Reference | -- | -- | -- |
| | Suburban | 0.15 | 0.03 | 0.76 | 0.02 |
| | Rural | 0.00 | 0.00 | Inf | 0.99 |
| | ICU Type | | | | |
| | Medical | Reference | -- | -- | -- |
| | Surgical | 1.75 | 0.23 | 13.16 | 0.59 |
| | Mixed/Other | 1.00 | 0.22 | 4.54 | 1.00 |
| | Teaching Hospital | 3.54 | 0.89 | 14.17 | 0.07 |
| | Hospital Size | | | | |
| | Small | Reference | -- | -- | -- |
| | Medium | 3.20 | 0.56 | 18.39 | 0.19 |
| | Large | 7.20 | 1.28 | 40.36 | 0.02 |

**eTable 2. Univariate Mixed Effects Logistic Regression Models of Association Between Patient Characteristics and Patient-Level Process Quality Measures (N=1536)**

| Process Quality Measure (Dependent Variable) | Patient Characteristic (Independent Variables) | Odds Ratio | 95% CI | | P value |
|---|---|---|---|---|---|
| Assessment of pain in last 24 hours of life | Male Sex | 0.97 | 0.64 | 1.45 | 0.86 |
| | Age | | | | |
| | 18-39 | Reference | -- | -- | -- |
| | 40-59 | 1.24 | 0.61 | 2.52 | 0.56 |
| | 60-79 | 2.40 | 1.14 | 5.05 | 0.02 |
| | ≥ 80 | 1.84 | 0.94 | 3.63 | 0.08 |
| | Race | | | | |
| | White | Reference | -- | -- | -- |
| | Black | 0.84 | 0.41 | 1.73 | 0.64 |
| | Asian | 0.57 | 0.19 | 1.74 | 0.33 |
| | Other | 0.55 | 0.27 | 1.10 | 0.09 |
| | Hispanic Ethnicity | 0.70 | 0.34 | 1.43 | 0.32 |
| | ICU Length of Stay (log transformed) | 1.21 | 0.92 | 1.59 | 0.17 |
| | Hospital Length of Stay (log transformed) | 1.23 | 0.94 | 1.60 | 0.14 |
| Offer or delivery of spiritual support during ICU stay | Male Sex | 0.97 | 0.76 | 1.26 | 0.85 |
| | Age | | | | |
| | 18-39 | Reference | -- | -- | -- |
| | 40-59 | 0.74 | 0.44 | 1.25 | 0.26 |
| | 60-79 | 0.79 | 0.47 | 1.34 | 0.38 |
| | ≥ 80 | 0.65 | 0.39 | 1.07 | 0.09 |
| | Race | | | | |
| | White | Reference | -- | -- | -- |
| | Black | 0.81 | 0.53 | 1.25 | 0.34 |
| | Asian | 0.56 | 0.29 | 1.10 | 0.09 |
| | Other | 0.98 | 0.62 | 1.56 | 0.95 |
| | Hispanic Ethnicity | 1.28 | 0.76 | 2.15 | 0.36 |
| | ICU Length of Stay (log transformed) | 1.34 | 1.13 | 1.60 | <0.01 |
| | Hospital Length of Stay (log transformed) | 1.30 | 1.10 | 1.54 | <0.01 |
| Assessment of delirium in last 24 hours of life[a] | Male Sex | 1.19 | 0.93 | 1.52 | 0.16 |
| | Age | | | | |
| | 18-39 | Reference | -- | -- | -- |
| | 40-59 | 1.01 | 0.62 | 1.64 | 0.98 |
| | 60-79 | 1.54 | 0.94 | 2.52 | 0.09 |
| | ≥ 80 | 1.69 | 1.05 | 2.71 | 0.03 |

© Kruser JM et al. *JAMA Network Open.*

| eTable 2 continued | | | | | |
|---|---|---|---|---|---|
| **Process Quality Measure (Dependent Variable)** | **Patient Characteristic (Independent Variables)** | **Odds Ratio** | **95% CI** | | **P value** |
| Assessment of delirium in last 24 hours of life | Race | | | | |
| | White | Reference | -- | -- | -- |
| | Black | 0.64 | 0.43 | 0.97 | 0.03 |
| | Asian | 0.53 | 0.27 | 1.04 | 0.06 |
| | Other | 1.24 | 0.80 | 1.93 | 0.33 |
| | Hispanic Ethnicity | 0.55 | 0.33 | 0.91 | 0.02 |
| | ICU Length of Stay (log transformed) | 1.10 | 0.94 | 1.30 | 0.23 |
| | Hospital Length of Stay (log transformed) | 1.10 | 0.94 | 1.29 | 0.24 |
| Ascertainment of advance directive during hospitalization | Male Sex | 0.90 | 0.71 | 1.14 | 0.38 |
| | Age | | | | |
| | 18-39 | Reference | -- | -- | -- |
| | 40-59 | 1.69 | 0.99 | 2.87 | 0.05 |
| | 60-79 | 2.17 | 1.28 | 3.69 | <0.01 |
| | $\geq$ 80 | 3.82 | 2.30 | 6.36 | <0.01 |
| | Race | | | | |
| | White | Reference | -- | -- | -- |
| | Black | 0.66 | 0.44 | 0.99 | 0.046 |
| | Asian | 1.08 | 0.56 | 2.09 | 0.81 |
| | Other | 0.84 | 0.55 | 1.28 | 0.41 |
| | Hispanic Ethnicity | 0.58 | 0.35 | 0.98 | 0.04 |
| | ICU Length of Stay (log transformed) | 1.07 | 0.91 | 1.25 | 0.42 |
| | Hospital Length of Stay (log transformed) | 1.09 | 0.94 | 1.27 | 0.27 |

© Kruser JM et al. *JAMA Network Open.*

## eTable 3. Comparison of Unit Characteristics Across Clusters

| Unit Characteristic; no. (%) | Cluster 1 n = 12[b] | Cluster 2 n = 25[b] | Cluster 3 n = 23[b] | P value[a] |
|---|---|---|---|---|
| ICU Type | | | | 0.65 |
| Medical | 3 (25) | 4 (17) | 3 (14) | |
| Surgical | 2 (17) | 1 (4) | 3 (14) | |
| Mixed Specialty/Other | 3 (58) | 18 (78) | 16 (73) | |
| Hospital Size | | | | 0.24 |
| Small | 4 (33) | 11 (50) | 4 (18) | |
| Medium | 5 (42) | 5 (23) | 9 (41) | |
| Large | 3 (25) | 6 (27) | 9 (41) | |
| Teaching Hospital | 8 (67) | 12 (50) | 18 (78) | 0.13 |
| Hospital Location | | | | 0.09 |
| Urban | 9 (75) | 10 (42) | 17 (74) | |
| Suburban | 3 (25) | 10 (42) | 3 (13) | |
| Rural | 0 (0) | 4 (17) | 3 (13) | |
| Geographic Location | | | | 0.62 |
| East Coast | 5 (42) | 8 (32) | 9 (39) | |
| Midwest | 2 (17) | 9 (36) | 9 (39) | |
| West Coast | 5 (42) | 8 (32) | 5 (22) | |
| Private Funding Structure | 7 (58) | 15 (63) | 16 (70) | 0.78 |
| Palliative Care Services Available | 11 (92) | 21 (91) | 19 (91) | |
| Admitting Structure | | | | 0.99 |
| Open | 5 (42) | 8 (33) | 6 (26) | 0.79 |
| Semi-Open | 3 (25) | 9 (38) | 7 (30) | |
| Closed | 4 (33) | 7 (29) | 10 (44) | |
| Clinicians Providing Coverage | | | | |
| Intensivist | 12 (100) | 21 (92) | 23 (100) | 0.21 |
| Residents | 7 (58) | 10 (46) | 17 (74) | 0.15 |
| Critical Care Fellows | 4 (33) | 6 (26) | 8 (35) | 0.80 |
| Advance Practice Providers | 8 (67) | 7 (30) | 17 (74) | 0.008 |

a: based on chi-squared
b: number of units per characteristic may not equal total number of units within cluster due to missing data

© Kruser JM et al. *JAMA Network Open.*

## eTable 4. Pairwise Comparisons of End-of-Life Event Rates Across Clusters

| Cluster Comparison | End-of-life Event | P value |
|---|---|---|
| 1 versus 2 | Extubated prior to death | <0.001 |
| 1 versus 2 | Absence of cardiopulmonary resuscitation in the last hour of life | 0.018 |
| 1 versus 2 | Delirium-free in the last 24 hours of life | <0.001 |
| 1 versus 2 | Pain-free in the last 24 hours of life | 0.004 |
| 1 versus 2 | Family or significant person(s) present at the time of death | 0.371 |
| 1 versus 3 | Extubated prior to death | <0.001 |
| 1 versus 3 | Absence of cardiopulmonary resuscitation in the last hour of life | 0.016 |
| 1 versus 3 | Delirium-free in the last 24 hours of life | <0.001 |
| 1 versus 3 | Pain-free in the last 24 hours of life | 0.005 |
| 1 versus 3 | Family or significant person(s) present at the time of death | 0.23 |
| 2 versus 3 | Extubated prior to death | 0.676 |
| 2 versus 3 | Absence of cardiopulmonary resuscitation in the last hour of life | 0.879 |
| 2 versus 3 | Delirium-free in the last 24 hours of life | <0.001 |
| 2 versus 3 | Pain-free in the last 24 hours of life | 0.81 |
| 2 versus 3 | Family or significant person(s) present at the time of death | 0.28 |

© Kruser JM et al. *JAMA Network Open.*

**eTable 5. Univariate Mixed Effects Logistic Regression Models of Association Between Patient-Level Process Quality Measures and EOL Events (N=1536)**

| End-of-life Event (Dependent Variable) | Patient-Level Process Measure (Independent Variable) | OR | 95% CI | P value |
|---|---|---|---|---|
| Receipt of cardiopulmonary resuscitation in last hour of life | Documentation of advance directive | 0.70 | 0.49-0.99 | 0.04 |
| | Offer of spiritual support | 0.76 | 0.53-1.07 | 0.12 |
| Extubated prior to death | Documentation of advance directive | 1.27 | 0.98-1.65 | 0.08 |
| | Offer of spiritual support | 1.26 | 0.96-1.65 | 0.09 |
| Family or significant person(s) present at time of death | Documentation of advance directive | 1.39 | 0.98-1.97 | 0.07 |
| | Offer of spiritual support | 1.95 | 1.37-2.77 | <0.01 |
| Delirium present in last 24 hours of life | Documentation of advance directive | 0.90 | 0.65-1.26 | 0.55 |
| | Offer of spiritual support | 0.66 | 0.45-0.96 | 0.03 |
| Pain present in last 24 hours of life | Documentation of advance directive | 1.30 | 0.99-1.70 | 0.06 |
| | Offer of spiritual support | 1.11 | 0.82-1.49 | 0.51 |

© Kruser JM et al. *JAMA Network Open.*

**eTable 6. Univariate Mixed Effects Logistic Regression Models of Association Between Unit-Level Structural Quality Measures and End-of-Life Events (N=1536)**

| EOL Event (Dependent Variable) | Unit-Level Structural Measure (Independent Variable) | OR (95% CI) | P value |
|---|---|---|---|
| Receipt of cardiopulmonary resuscitation in last hour of life | Continuity of Care Policy | 0.87 (0.55-1.38) | 0.57 |
| | Open Visitation Policy | 0.64 (0.39-1.06) | 0.08 |
| | Protocol for Withdrawal of MV at EOL | 0.74 (0.48-1.14) | 0.17 |
| | Other protocols for EOL care | 0.99 (0.60-1.65) | 0.98 |
| | Staff Reflection Opportunity | 0.89 (0.54-1.47) | 0.65 |
| Extubated Prior to Death | Continuity of Care Policy | 0.79 (0.55-1.15 | 0.22 |
| | Open Visitation Policy | 1.14 (0.73-1.78) | 0.57 |
| | Protocol for Withdrawal of MV at EOL | 1.31 (0.89-1.91) | 0.17 |
| | Other protocols for EOL care | 1.14 (0.73-1.77) | 0.57 |
| | Staff Reflection Opportunity | 0.76 (0.51-1.14) | 0.18 |
| Family or significant person(s) present at time of death | Continuity of Care Policy | 1.61 (0.96-2.70) | 0.07 |
| | Open Visitation Policy | 1.24 (0.66-2.30) | 0.51 |
| | Protocol for Withdrawal of MV at EOL | 1.31 (0.77-2.21) | 0.31 |
| | Other protocols for EOL care | 1.22 (0.67-2.19) | 0.51 |
| | Staff Reflection Opportunity | 1.09 (0.61-1.93) | 0.78 |
| Delirium present in last 24 hours of life | Continuity of Care Policy | 1.03 (0.45-2.35) | 0.94 |
| | Open Visitation Policy | 1.19 (0.42-3.33) | 0.74 |
| | Protocol for Withdrawal of MV at EOL | 0.78 (0.34-1.76) | 0.54 |
| | Other protocols for EOL care | 1.31 (0.53-3.25) | 0.55 |
| | Staff Reflection Opportunity | 0.51 (0.21-1.23) | 0.13 |
| Pain present in last 24 hours of life | Continuity of Care Policy | 0.79 (0.46-1.35) | 0.39 |
| | Open Visitation Policy | 2.21 (1.15-4.27) | 0.02 |
| | Protocol for Withdrawal of MV at EOL | 1.64 (0.97-2.79) | 0.07 |
| | Other protocols for EOL care | 1.21 (0.65-2.24) | 0.53 |
| | Staff Reflection Opportunity | 1.05 (0.58-1.87) | 0.88 |

Abbreviations: EOL = end of life; MV = mechanical ventilation

© Kruser JM et al. *JAMA Network Open.*
